# A prospective cohort study to evaluate immunosuppressive cytokines as predictors of viral persistence and progression to pre-malignant lesion in the cervix in women infected with HR-HPV: study protocol

**DOI:** 10.1186/s12879-018-3490-1

**Published:** 2018-11-19

**Authors:** K. Torres-Poveda, M. Bahena-Román, K. Delgado-Romero, V. Madrid-Marina

**Affiliations:** 1 Chronic Infectious Diseases and Cancer Division, Center for Research on Infectious Diseases, Instituto Nacional de Salud Pública (INSP), Cuernavaca, Morelos Mexico; 2 CONACYT-INSP, Cuernavaca, Morelos Mexico; 3 Centro de Atención para la Salud de la Mujer (CAPASAM) (Center for Women’s Health), Health Services of the State of Morelos, Cuernavaca, Mexico

**Keywords:** HPV, Viral persistence, Cytokines, Cohort, Mexico

## Abstract

**Background:**

Cervical cancer (CC) is caused by a persistent infection of high-risk human papillomavirus (HR-HPV). While most HPV infections are transient, persistent HPV infections are a significant health problem in Mexico. With an estimated HPV prevalence of 10% among women in reproductive age, approximately 25% of these women present at least a positive result in triage test, which according to previous studies is expected to be confirmed as positive CIN-2/3. The immune system has a key role in the natural history of HPV infection; alterations in the cellular immune response are responsible for the failure to eliminate HPV. The objective of this project is to assess the prognostic value of detecting immune markers (IL-10, IL-4, TGFβ1, IFNγ, IL-6, and TNFα), the expression of HPV-HR E6/E7 proteins, and the viral load at the cervical level with respect to the persistence or clearance of HR-HPV infection, and the regression or progression of a cervical premalignant lesion.

**Methods:**

A dynamic cohort study is being conducted in women with colposcopic, cytological, and histopathological results negative for squamous intraepithelial lesion (SIL) in the cervix and a positive HPV test; the subjects will be followed-up for 5 years, period from which 3 years have already elapsed, with yearly studies (colposcopy, cytology, and histopathology diagnosis, along with molecular HPV test, quantification of viral load and of IL-10, IL-4, TGFβ1, INFγ, IL-6, and TNFα levels, along with the expression of the HR-HPV E6/E7 proteins in the cervix as a viral marker. The outcome will be categorized as viral persistence or clearance; and as SIL persistence, progression, or regression. Binomial and/or multinomial regression models adjusted for potential confounders will be used, associating the relative risk of the outcome with the immune and viral markers evaluated.

**Discussion:**

This research will generate knowledge about immune markers with predictive value for the persistence and clearance of HPV, which will improve the triage of positive HPV women and thus reduce the economic burden for the Mexican health system imposed by the management of high-grade SIL and CC cases, which are still detected in late stages.

## Background

Cervical cancer (CC) is caused by a persistent infection by high-risk human papilloma virus (HR-HPV). The prevalence and estimated role of different HPV genotypes in the development of cervix premalignant lesions and CC has been described in several meta-analyses [1, 2]. In one of the first prospective studies on the prevalence of oncogenic HPV, a concordant pattern relating HPV prevalence with younger age, viral elimination, and the reduction of exposure to new types of HPV was reported [3]. Viral prevalence is the product of incidence (acquisition of new infections) and duration (persistence). Factors that could favor a higher HPV prevalence include age, female or male sexual behavior, increased detection of HPV infection, changes in the cervicovaginal epithelium due to age or related to the menopause, and age-related immune senescence, which leads to the reactivation of latent infections and thus to an apparent increase in the detection of new infection cases [3].

A persistent HR-HPV infection has been considered as a prerequisite for CC development [4]. In most studies, viral persistence is defined as the detection of the same HPV type or the same type-group in two consecutive visits; however, the inter-visit period ranges from 4 months to 5–7 years [3, 5–7]. Thus, some studies suggest restricting the analysis to incidental infections, taking into account the duration of the infection instead of the number of positive tests [5, 8]. An agreement on the definition of viral persistence would facilitate making comparisons of the results in studies of this type and would provide a guide to define the endpoints to be evaluated in clinical trials of HPV vaccines and to make recommendations to improve the CC screening policies.

The reasons why some HPV infections are cleared whilst others persist, increasing the high risk of squamous intraepithelial lesions (SIL), are still under study. The mechanistic explanation of HPV elimination relies on specific immunological reactions, where competent cellular and humoral responses are both required [9]. The immune system plays a key role in the natural history of HPV infection; alterations in the cellular immune response are responsible for the failure to eliminate HPV [10]. On the other hand, immune tolerance has been reported to favor viral persistence and progression to cancer [11].

Highlighting the relevance of acquired immunity in women with HPV infection, a higher SIL incidence has been observed in immunocompromised patients [12]. While an impaired cellular immunity hinders the clearance of HPV infection, 2% of HR-HPV infections persist in apparently immunocompetent individuals [13].

Many molecular alterations in those women who progress to CC have been described, the most common being immunosuppression produced by Th2 (IL-4, IL-6, IL-10) and Th3 (TGFβ1) cytokines [14, 15]. Patients with HPV-associated neoplasia showing a Th1 cytokine profile had a better clinical outcome compared to those patients exhibiting a Th2 profile [12]. CC progression has been associated with an undesirable Th1- to Th2-cytokine type shift induced by two E7-derived epitopes and an increase in IL-10 expression [16].

The role of IL-10 and TGFβ1 in the immunosuppression observed in cervix SIL and CC patients has been described [17–19]. IL-10 is a potent immunosuppressive cytokine that favored viral persistence in a model of persistent cytomegalovirus infection [20]. HPV regulates the expression of these cytokines, and both the HPV-16-derived E6 and E7 proteins have been reported to increase the expression of TGFβ1, while the HPV-derived E2 protein regulates the expression of IL-10 [17, 21].

Some genetic risk determinants of persisting HPV infection have been defined [22], including polymorphisms in genes controlling effector T cell responses [13]. Most association studies for CC around the world evaluate single nucleotide polymorphisms (SNPs) in candidate genes involved in oncogenesis and the cellular immune response, since there is much evidence of viral evasion to the immune response in patients with persistent HPV infection and CC [19, 23, 24].

Several studies have reported an association between CC and polymorphisms in the promoter region of IL-10, IL-4, IL-6, and TGFβ1, showing an increased expression of these cytokines in the cervix; based on this evidence, a genetic immune profile (Th2-Th3) was postulated as underlying the susceptibility to CC [25]. However, this profile was defined in cross-sectional studies, and a cohort study is required to determine whether this genetic profile actually favors viral persistence.

Previous studies on HPV persistence evaluating the immune response were made after viral persistence was established, so it was not possible to determine whether immune dysregulation leads to HPV persistence or vice versa. Therefore, follow-up studies such as the one proposed in this protocol are required to address the question on whether the persistence of HPV infection is the cause or the consequence of immune dysregulation. On the other hand, immunological predictors are much needed to identify women with potential for viral persistence and progression to SIL, alleviating the current overload in diagnosis and treatment services, improving thus the CC prevention strategy.

## Methods/design

### Objectives

The general objective of this project is to assess the prognostic value of detecting immune markers (IL-10, IL-4, TGFβ1, IFNγ, IL-6, and TNFα) at the systemic and cervical levels, either coupled or not with the expression of HR-HPV E6/E7 proteins and viral load in the cervix, with respect to the persistence or clearance of HPV infection and to the incidence, regression, or progression of SIL in cervix.

To achieve this, a baseline study will be conducted, and risk groups will be generated by determining the frequency of HPV genotypes. The levels of the immune biomarkers IL-10, IL-4, TGFβ1, IFNγ, IL-6, and TNFα; the levels of the oncoproteins E6 and E7; and the viral load at the level of cervix, will also be determined.

For the follow-up of the cohort, changes in the levels of the immune biomarkers IL-10, IL-4, TGFβ1, IFNγ, IL-6, TNFα, the oncoproteins E6 and E7, and the viral load will be determined in the cervix of the subjects at fixed times. The number of new cases of persistence and viral clearance, along with the incidence of cervix SIL regression or progression will be determined.

To assess the final event of the cohort, the relative risk for possible persistence and viral clearance adjusted by confounders with respect to high levels of each immune biomarker in the cervix will be estimated, as well as with respect to high levels of the proteins E6 and E7, and to viral load at the cervical level.

The relative risk adjusted for possible confounders of incidence, regression or progression of SIL in cervix with respect to high levels of each immune biomarker in the cervix will also be evaluated, as well as with respect to high levels of the proteins E6 and E7 and to viral load at the cervical level. Finally, the interaction between cervical levels of each immune marker and the viral load, and its association with HPV persistence, will be evaluated.

### Design and population

A prospective, dynamic cohort study will be conducted on women attending the Women’s Health Care Center—CAPASAM of the Health Services of the State of Morelos, Mexico, after an abnormal result in cytology studies recently issued by a referring health center.

### Inclusion and exclusion criteria

All women attending the CAPASAM are eligible for recruitment if they: (1) are 30 years-old or older; (2) have resided for 5 years or more in Morelos; (3) agree to participate in the study, sign an informed consent form, complete a questionnaire, and donate blood and cervix exudate samples; (3) have a negative diagnosis for chronic inflammatory or autoimmune diseases at the beginning of the study and during follow-up, and a negative pregnancy status; (4) received no previous anti-HPV vaccination, nor immunosuppressive treatment within 6 months before their inclusion in the cohort; and (5) provide at least a telephone number to be contacted. Those individuals for whom follow-up is impossible and those who decide to stop participating in the study at any time will be excluded.

### Recruitment

Recruitment started in September 2015, and it is planned to continue until 2020. A unique study identification number is assigned to each woman. This number identifies and tracks the questionnaires and biological samples collected during the study to make them reversibly anonymized. This procedure is fundamental to link the collected information, ensure confidentiality, and protect personal data in accordance to Mexican laws.

### Sample size

Assuming a power of 90% and a confidence level of 95%, a sample size of the risk groups to be followed-up in the cohort with an estimated loss of 20% during the follow-up period was calculated, taking into account the mean serum levels (pg/mL) and the respective standard deviation for each cytokine to be evaluated as immune marker, obtained in a previous cross-sectional study in HPV-positive patients with no SIL and HPV-positive women with SIL in cervix [25]. Thus, the inclusion of 200 HPV-positive women was determined as necessary. Calculations were performed with Stata v. 14.0 (StataCorp, College Station, TX, USA).

### Sampling and follow-up

**Fig. 1:**
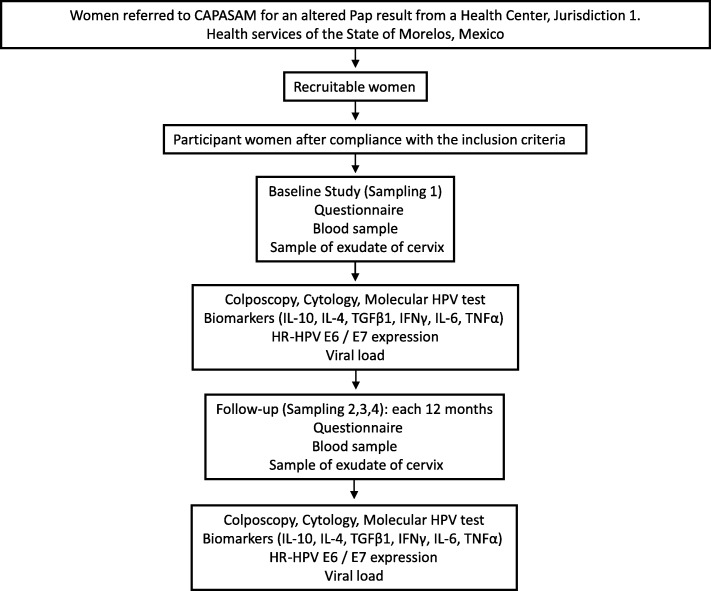
Assessment and follow-up of the cohort

All included subjects will be evaluated and followed-up for 5 years (Fig. 1). At the baseline (sampling 1), a structured questionnaire will be given to each subject and the information in each questionnaire will be entered in a database. The software Stata v.14.2 will be used for this. From each participant who has signed the informed consent form and completed the questionnaire, a blood sample will be taken by a healthcare professional, using EDTA-containing Vacutainer tubes (7 mL) and serum-separator Vacutainer tubes (4 mL) (Becton Dickinson, BD Franklin Lakes, NJ, USA). A CAPASAM-ascribed colposcopic gynecologist will take three exudate samples from the endocervical canal; the first sample will be collected by applying an ophthalmic sponge for 30 s, removing the device from the patient, and immediately placing it in a cryovial and into liquid nitrogen to measure cytokine levels in the cervix; approximately 10 min later, a second sample will be taken with an exfoliative brush (cytobrush) and placed in a tube with preservation medium to measure the expression levels of the HPV oncoproteins E6 and E7; finally, a third sample will be taken using the ThinPrep HOLOGIC vial for automated HPV genotyping.

In addition, the gynecologist will make a colposcopic diagnosis at the consultation time, will take a sample for a cytology or Papanicolaou study, and will send it for pathological analysis to the CAPASAM Pathology Unit. Any potential risk and discomfort during sampling are clearly described in the informed consent form. Four follow-up samplings will be performed, one every 12 months through the five-year study. For follow-up samplings, the patients will be contacted by telephone and invited to attend the CAPASAM to perform each programmed blood and cervix exudate sampling. Additionally, variables of the gynecological-obstetric history and sexual behavior that may have changed over time, such as marital status and number of sexual partners, will be recorded.

## Variables

### Dependent variables

HPV persistence, understood as the detection of the same HPV genotype in two or more consecutive intervals.

Viral clearance, understood as the negative detection of the same genotype in a consecutive interval, following a positive sample (first negative PCR result after an incidental infection).

SIL persistence: histopathological diagnosis of (low- or high-grade) SIL after a first SIL diagnosis. Persistence of the same lesion in two or more successive liquid cytologic studies.

SIL regression: histopathological diagnosis for SIL negative after a first (low- or high-grade) SIL diagnosis. Regression of the lesion in two or more successive cytologic studies and subsequent healing.

Progression of (low- or high-grade) SIL: histopathological diagnosis of high-grade SIL or cancer in situ after a first diagnosis of SIL (low- and high-grade, respectively).

**Main independent variable:** levels at the cervix of the immune biomarkers IL-10, IL-4, TGFβ1, IFNγ, IL-6, and TNFα, expressed in pg/mL.

### Other independent variables

Expression levels of the proteins E6 and E7 in the cervix, reported in terms of expression over the endogenous glyceraldehyde-3-phosphate dehydrogenase (GAPDH) gene. Viral load in cervix, reported as the number of viral copies. Age, number of sexual partners, age at menarche, age at start of active sex life, socioeconomic level, parity, smoking (years, number of cigarettes per day, whether the habit is old or current, previous years of tobacco use), hormonal contraception (years of duration, current situation), viral genotype, history of other sexually transmitted infections (STIs), conservative clinical treatment for previous SIL and duration of an incidental HPV infection, understood as the time elapsed from the onset of incidental infection in the cohort until its elimination, assuming that both events occurred in the inter-visit period, observed as a shift in HPV status (i.e. from negative to positive or from positive to negative).

### DNA extraction from cervical exudate

When cervical exudate samples were taken with an exfoliative brush (cytobrush), efforts will be made to recover most of the cells in the brush; the supernatant will be centrifuged at 5000 rpm for 3 min. Cells recovered from the cytobrush will be added with 400 μL of lysis solution. After incubating at 65 °C for 5 min, 600 μL of chloroform will be added, mixed by inversion five times, and centrifuged at 10 000 rpm for 4 min; the upper phase will be transferred to a new tube, and 800 μL per sample of freshly prepared precipitation solution (80 μL of concentrated precipitation solution plus 720 μL of sterilized water) will be added; the tube will be mixed by inversion for 40 s and centrifuged at 10 000 rpm for 4 min; the supernatant will be discarded and the DNA tablet will be dissolved in NaCl solution and homogenized 3–4 times; then, 300 μL of absolute ethanol will be added and the tube will be incubated at − 20 °C for 10–20 min and centrifuged at 10 000 rpm for 4 min; the supernatant will be discarded, and 1 ml of 70% ethanol will be added; after centrifuging at 10 000 rpm for 5 min, the supernatant will be discarded and the DNA pellet will be dissolved in 50 μL of sterile water. When cervical exudate samples were taken with the Merocel ophthalmic sponge, the extraction will be performed according to a previously described protocol [26]. DNA concentration and purity will be assessed in a Thermo Scientific NanoDropTM 1000 Spectrophotometer (260/280) and the integrity of the DNA will be determined by electrophoresis in 0.8% agarose gels.

### RNA extraction from cervical exudate

Per 6 × 10^6^ cells or 50–100 mg of sample, 1 ml of Trizol will be added. The sample will be homogenized and incubated for 5 min at room temperature. Then, 200 μL of chloroform per milliliter of Trizol will be added, mixed, and incubated for 2–3 min at room temperature. The tube will be centrifuged at 12 000 rpm for 15 min at 8 °C or 10 min at 4 °C. The upper phase, containing RNA, will be transferred to a tube; 500 μL of isopropanol will be added, mixed, and incubated at − 20 °C for 15 min; after centrifuging at 12 000 rpm for 10 min at 4 °C, the supernatant will be removed. The RNA pack will be washed with 1 mL of 75% ethanol, mixed and centrifuged at 10 000 rpm for 7 min at 4 °C; the supernatant will be discarded, and the tablet will be resuspended in 20 μL of RNase- and DNase-free water. Then, the sample will be incubated at 65 °C for 10 min, and RNA will be quantified and stored at − 80 °C until used for real time RT-PCR assays to analyze the expression of the cytokines IL-10, IL-4, TGFβ1, IFNγ, IL-6, and TNFα, and to evaluate the expression of the HPV E6 and E7 oncoproteins.

### Analysis of cytokine expression and of HPV E6 and E7 oncoprotein expression at the cervical level

Total RNA isolated from cervical exudate will be used to synthesize cDNA; this will be performed in the presence of 200 U of reverse M-MLV transcriptase and 2.5 μg of total RNA under standard conditions. PCR will be carried out in a reaction volume of 25 μL containing 1 μL of cDNA, dNTPs 0.2 mM, 15 pmol of each oligonucleotide, 2.5 μL of reaction buffer and 1 U of recombinant Taq DNA polymerase. The constitutive gene of GAPDH (250 bp) will be used to verify DNA integrity. PCR will be carried out in a Mastercycler PCR gradient thermocycler (Eppendorf, Germany) under the following conditions: 5 min at 94 °C, 35 one-minute cycles at 94 °C, 1 min at 60 °C, and 1 min at 72 °C, with a final extension step of 10 min at 72 °C. Amplification products will be resolved by electrophoresis in a 6% polyacrylamide gel and visualized under ultraviolet light after staining with ethidium bromide.

Expression probes to analyze the expression of the cytokines IL-10, IL-4, TGFβ1, IFNγ, IL-6, IL-2, and TNFα and the expression of the HPV E6 and E7 oncoproteins will be obtained from Applied Biosystems for real-time PCR analysis. The HPRT1 (hypoxanthine phosphoribosyl transferase) gene will be used to normalize the amount of mRNA in each sample to analyze IL-10, IL-4, TGFβ1, IFNγ, IL-6, and TNFα. The GAPDH gene will be used to normalize the amount of mRNA in each sample to analyze the expression of the HPV E6 and E7 oncoproteins.

Real-time PCR will be performed by adding 2 μL of each cDNA sample to a final reaction volume of 10 μL, containing 5 μL of Master Mix for expression, 0.4 μL of probe, and 2.6 μL of molecular grade, DNase-free water. Amplification cycles will be carried out in an Applied Biosystems VIA-VII equipment (Foster City) under the following conditions: 10 min at 94 °C, 40 one-minute cycles at 94 °C, 1 min at 54 °C, and 1.5 min at 72 °C, with a final extension step of 15 min at 72 °C. The level of mRNA expression for the genes under study will be calculated by relative quantification with the comparative Ct method (2-ΔCt) and plotted as expression relative units of each gene relative to the endogenous gene (HPRT-1 or GAPDH) and to the group of comparison. All samples will be analyzed in duplicate.

### Cytokine evaluation in cervical secretions

The levels of IL-10, IL-4, TGFβ1, IFNγ, IL-6, and TNFα will be determined by the Luminex Multiplex assay, according to a previously described protocol [27].

### HPV detection and genotyping in cervical exudate samples

Samples taken in ThinPrep (Pap test) vials from each subject who agreed to participate in the cohort study will be stored at 4 °C until use for HPV genotyping by the automated Cobas HPV Test. The Cobas 4800 is a fully automated real-time PCR system that separately detects HR-HPV-16 and -18 genotypes in addition to ten other high-risk genotypes (31, 33, 35, 39, 45, 51, 52, 56, 58, 59) and two of “probable high risk” (66 and 68). The procedure will be performed according to Roche Diagnostic’s instructions.

### Viral load quantification

Viral load in the cervix will be evaluated with the Seegene HPV 28 Anyplex system, using DNA extracted from cervix exudate from each patient. This will allow us to confirm HPV diagnosis and at the same time to estimate the viral load by genotype in each patient.

### Data collection and statistical analysis

A descriptive analysis of the sociodemographic and gynecological-obstetric characteristics, familial history of cancer, and lifestyle-related variables in the population under study will be performed. The questionnaire will include, among other variables: sociodemographic characteristics such as age, marital status, religion, education level, smoking habit (years, number of cigarettes per day, whether the habit is old or current, previous years of tobacco use), and socioeconomic level; for this variable, an index (low, medium, and high tertiles) will be constructed using the analysis of main components for the population included in the cohort, with information on household floor materials and availability of tap water, washing machine, refrigerator, television, radio and stove; gynecological-obstetric traits such as the number of sexual partners, regular partners (defined as sexual activity with that person for at least 6 months), age at menarche, age at start of active sex life, parity, hormonal contraception (years of duration, current situation), history of sexually transmitted diseases, condom use, genital hygiene, previous HPV infection, previous local treatment for a cervical lesion, and familial history of cancer, including type of cancer and consanguinity.

For continuous variables, expressed as a mean ± standard deviation, the Kruskal-Wallis test will be used. For categorical variables, expressed as a percentage, the chi-square test will be used. Results will be regarded as statistically significant for *P* < 0.05. All data will be analyzed with STATA v.14 for Windows. Missing data will be addressed by using Maximum likelihood, multiple imputation, and inverse probability weighting and analyzed via multilevel mixed-effects linear regression models.

To evaluate the levels of the immune biomarkers IL-10, IL-4, TGFβ1, IFNγ, IL-6, and TNFα and the expression levels of the proteins E6 and E7 in the cervix, the non-parametric Mann-Whitney U test will be used. A curve of diagnostic performance (ROC-receiver operating characteristic) will be plotted to obtain the cut-off point of greater discrimination with respect to the variable of evolution (viral persistence, viral clearance, SIL incidence, SIL persistence, SIL progression. Once obtained, the sensitivity, specificity, positive predictive value, and negative predictive value for the cut-off points of each variable will be calculated. With respect to viral load, the Wilcoxon rank sum test will be used to measure the difference in median viral load for each HPV type, according to the infection status (HPV persistence or HPV clearance). All possible 2-way interactions between cervical levels of each immune marker and the viral load, and its association with HPV persistence, will be evaluated by adding multiplicative terms in the multivariate logistic models.

The incidence and cumulative incidence rate of each outcome (HPV persistence, HPV clearance, SIL incidence, SIL persistence, SIL progression) will be determined, adjusting for changing levels of the immune biomarkers IL-10, IL-4, TGFβ1, IFNγ, IL-6, and TNFα and the expression levels of E6 and E7 in the cervix.

To assess the association of viral persistence or clearance, SIL incidence, regression, or progression with cervical levels of the immune biomarkers IL-10, IL-4, TGFβ1, IFNγ, IL-6, and TNF, as well as with E6 and E7 expression levels and with cervical viral load, a Cox regression analysis will be performed, adjusting for the co-variables age, number of sexual partners, age at menarche, age at start of active sex life, parity, smoking, hormonal contraception, history of other STIs, conservative clinical treatment, viral genotype, co-infection with two or more HPV genotypes, and duration of incidental HPV infections. The viral load will be included in the analysis as the maximum viral load reached during an incidental infection for each viral group. The endpoints for the analysis will be persistence or viral clearance and SIL incidence, regression, or progression.

The Kaplan-Meier method will be used to estimate the median duration of infection for most HPV types and for each previously defined viral group. Infections will be considered as persistent when their duration is longer than the median duration of the infection. A longitudinal approach will be applied to cluster all possible triplets of consecutive visits per individual, to compare the results of this measure of persistence with that obtained by using the traditional persistence definition (i.e. two consecutive positive samples).

To evaluate the association of persistent infection with the risk to develop cervical intraepithelial neoplasm (CIN) grade-1 or grade-2/3, a Cox regression analysis will be performed, adjusting for cofactors relevant to the infection, like smoking and co-infection, along with the levels of the immune biomarkers IL-10, IL-4, TGFβ1, IFNγ, IL-6, and TNFα, the expression levels of E6 and E7 and the viral load in the cervix, and co-infection with two or more HPV genotypes. The endpoints for the analysis will be the histopathological diagnosis of CIN 1, CIN 2, CIN 3, or carcinoma in situ.

According to the results of HPV genotyping, type-specific viral clearance rates in single and multiple infections will be compared with the stratified log-rank test. This test will also determine the probability of viral clearance among HPV variants. The effect of co-infection by HPV genotype on incidence rates for CIN 1 and CIN 2/3 in women with simple and multiple infections will be assessed by the Cochran Mantel-Haenszel test stratified by age, the result of cytologic studies, and HPV type.

## Discussion

The worldwide prevalence of HPV infection in women without cervix abnormalities is 11–12%, with the highest rates reported in sub-Saharan Africa (24%), Eastern Europe (21%), and Latin America (16%). The two most frequent viral types are HPV-16 (3.2%) and HPV-18 (1.4%). Prevalence increases in women with cervical pathology in direct proportion to the severity of the lesion, approaching to 90% in women with CIN 3 and invasive cancer [28, 29]. Most HPV infections are transient and intermittent. The immune system plays an important role in the natural history of HPV infections, since most high-risk HPV infections (90%) [30], as well as most low-grade intraepithelial lesions (75%) are eliminated [31, 32]. However, if the infection with a high-risk HPV type persists, viral genes can interfere with the cellular control mechanisms and trigger neoplastic changes, which eventually could progress to an invasive carcinoma [33].

The natural history of CC has been established by several prospective cohort studies, and the factors involved in the regression, persistence, and progression of cervical lesions are well known [9]. The outcome of HPV infections follow a well-characterized pattern where a dynamic balance is set between incidental infections and virus clearance. A rapid accumulation of incidental infections once sex activity starts (women younger than 20 years of age) is followed by a shift in this balance after the subjects are 25 years old, to favor virus clearance. This explains the steadily declining age-specific prevalence of HPV infections until menopause [9].

However, various aspects of the dynamics of HPV infection are still poorly understood [34, 35]. In particular, the mechanisms of virus clearance are controversial [35]. The importance of HPV clearance and persistence has been recognized for some years, and the number of studies addressing these issues has increased substantially [9]. The clearance of HPV infection is usually attributed to an effective immune response, and the observation of longer clearance times in immunocompromised individuals further corroborates this assumption [36, 37].

A wide variety of variables have been explored as potential co-determinants and/or predictors of HPV clearance; however, this is a yet largely unexplored area [9]. The likelihood of an HPV infection to become persistent and progress into invasive CC should be seen as the result of the combined effect of certain viral- (HPV genotype) and host-dependent features (the immune status of the subject) [12].

Direct evidence linking host immunologic responses to the risk of HPV persistence is sparse. The few studies that have been published to date have been modest in size. Therefore, it is not surprising that the results have been mixed. While some studies have suggested that the immune response to HPV is associated to viral clearance, others have concluded otherwise [9]. Further studies on host immunologic factors associated with HPV persistence in well-characterized, larger studies are clearly needed [22].

Given that a persistent infection by HR-HPV may be regarded as an intermediate phenotype and is a reliable predictor of CC, this study is expected to have a scientific impact since it will establish the prognostic value of detecting immune markers (IL-10, IL-4, TGFβ1, IFNγ, IL-6, and TNFα), the expression of HR-HPV E6/E7 proteins, and the cervical viral load with respect to the persistence or clearance of HPV infection and the incidence, regression, or progression of SIL in cervix, which can be evaluated at the population level and used to monitor and prevent the progression of this intermediate phenotype to CC.

The immune markers with potential predictive value for HPV persistence or clearance studied in this project will improve the triage of positive HPV women and thus reduce the costs imposed by the management of high-grade premalignant lesions and CC, which are still detected in later, advanced stages.

Although measuring viral persistence has a prognostic value and is useful to understand the natural history of HPV infection and SIL, it is necessary to study additional viral variables to improve risk assessment. In this study, we propose to evaluate the expression of E6 and E7 and the viral load at the cervical level. HPV viral load has been reported as an auxiliary marker of persistent HPV infection; since the biological behavior of the various HPV types differ, the predictive value for viral persistence of the HPV DNA load can also vary among HPV types [38]. Therefore, the relationship of the type-specific HPV viral load with the clearance or persistence of HPV infection and the incidence, regression, or progression of SIL will also be explored in this study.

The limitations of this research project are those inherent to a cohort study; for instance, losses in follow-up could arise as this project develops, since patients can leave the study either due to a lack of interest or a change of residence; likewise, additional losses in follow-up may occur due to pregnancy or a disease compromising the immune response of the patients being monitored. A second limitation is the possibility of information biases, since although the interview will be conducted by qualified personnel, the patients may not remember accurately the information required for the study.

## Abbreviations

CC: Cervical cancer
CIN: Cervical intraepithelial neoplasm
GAPDH: Glyceraldehyde-3-phosphate dehydrogenase
HPRT1: Hypoxanthine phosphoribosyl transferase
HR-HPV: High-risk human papilloma virus
IFNγ: Interferon-gamma
IL-10: Interleukin 10
IL-4: Interleukin 4
IL-6: Interleukin 6
RT-PCR: *Reverse-transcription* polymerase chain reaction
SIL: Squamous intraepithelial lesions
SNPs: Single nucleotide polymorphisms
STIs: Sexually transmitted infections
TGFβ1: Transforming growth factor beta 1
Th: T helper cells
TNFα: Tumor necrosis factor alpha
